# Subcutaneous and Sublingual Immunotherapy in Allergic Asthma in Children

**DOI:** 10.3389/fped.2017.00082

**Published:** 2017-04-21

**Authors:** Sophia Tsabouri, Antigoni Mavroudi, Gavriela Feketea, George V. Guibas

**Affiliations:** ^1^Child Health Department, University of Ioannina School of Medicine, Ioannina, Greece; ^2^Allergy Unit of the 3rd Pediatric Department, Aristotle University of Thessaloniki, Thessaloniki, Greece; ^3^General Hospital of Ilias, Amaliada Hospital Unit, Amaliada, Greece; ^4^Division of Infection, Immunity and Respiratory Medicine, University of Manchester, Manchester, UK; ^5^Allergy Department, University Hospitals South Manchester NHS Trust, Manchester, UK

**Keywords:** subcutaneous immunotherapy, sublingual immunotherapy, children, allergy, asthma

## Abstract

This review presents up-to-date understanding of immunotherapy in the treatment of children with allergic asthma. The principal types of allergen immunotherapy (AIT) are subcutaneous immunotherapy (SCIT) and sublingual immunotherapy (SLIT). Both of them are indicated for patients with allergic rhinitis and/or asthma, who have evidence of clinically relevant allergen-specific IgE, and significant symptoms despite reasonable avoidance measures and/or maximal medical therapy. Studies have shown a significant decrease in asthma symptom scores and in the use of rescue medication, and a preventive effect on asthma onset. Although the safety profile of SLIT appears to be better than SCIT, the results of some studies and meta-analyses suggest that the efficacy of SCIT is better and that SCIT has an earlier onset than SLIT in children with allergic asthma. Severe, not controlled asthma, and medical error were the most frequent causes of SCIT-induced adverse events.

## Introduction

Asthma is one of the most common chronic inflammatory disorders in children, and airway remodeling can cause it to persist into adulthood. It affects up to 300 million people worldwide, and it is believed that an additional 100 million people will be suffering with asthma by 2025 (1). It has been shown that there are numerous asthma phenotypes from infancy to adulthood. Although asthma is not exclusively associated with allergy/atopy, about 75% of all children with asthma are atopic (1).

It is known that asthma pharmacotherapy can effectively control symptoms and the ongoing inflammatory process. However, it can not affect the underlying immune response; when medication is discontinued, symptoms may recur. This is where allergen immunotherapy (AIT) comes into play, as the only management that can interfere with the underlying immune pathophysiology. AIT is recommended for patients with moderate to severe allergic rhinitis with/without mild to moderate asthma due to inhalant allergens (2, 3). AIT is the only therapeutic method that may alter the natural course of allergy affecting both the development of new sensitizations and the clinical disease development (including deterioration of symptoms and progression of rhinitis to asthma) (4). The predominant mechanism is dependent on the type of allergen-specific T_H_ cells (5). The efficacy of both subcutaneous subcutaneous immunotherapy (SCIT) and sublingual immunotherapy (SLIT) has been shown by systematic reviews and meta-analyses for both perennial and seasonal allergic respiratory disease (4, 6). However, the clinical evaluation of AIT must take into account the high heterogeneity among studies. Nevertheless, the Global Initiative for Asthma Report has been updated in 2017 and stated that potential benefits of AIT, compared to pharmacological and avoidance options, must be weighed against the risk of adverse effects, and the inconvenience and cost of the prolonged course of therapy (7).

The objective of the current review is to summarize the evidence for the efficacy, safety, potential barriers to, and facilitators of the use of AIT in pediatric asthma.

## Definition

Subcutaneous immunotherapy is the term used to describe a process of repeated doses of a specific relevant allergen, for the treatment of IgE-mediated allergic disease (8). The conventional schedule for SCIT that employs unmodified allergen extracts consists of a weekly dose buildup by subcutaneous injections, followed by maintenance doses at 4 or 8 week intervals. Fewer buildup doses are possible with the use of modified allergenic extracts (such as allergoids), and/or adjuvants (9).

Sublingual immunotherapy is an alternative approach of allergen immunotherapy, whereby allergens are administered orally and—more specifically—by the sublingual route. In SLIT the allergen is given as either a dissolvable tablet or an aqueous/liquid extract (10), and the time interval between each maintenance dose varies from one product to another; generally, the once-a-day administration is preferred (11).

## History

### History of SCIT

In 1911, Dr. L. Noon and Dr. J Freeman published their findings on allergy desensitization through subcutaneous injections of pollen extract. By 1935, Cooke and colleagues identified a protective factor in serum, which was induced by AIT. This finding led to the concept of “blocking antibodies” (12). In 1953, Johnstone and Dutton randomized all children attending their clinic to receive either treatment (higher doses of SCIT) or placebo. The asthma symptoms of the treatment group resolved after 4 years (12).

After the second World War, aluminum hydroxide (Alum) was used as adjuvant in most allergen preparations. In the last decades, other modalities were tried, showing promising results (13). Other modifications also took place such as the use of inactivated allergoids in order to reduce their ability to bind to IgE, while retaining their ability to stimulate immune responses (12). In 1986, however, concerns were raised in the UK regarding the safety of desensitization, as several severe reactions had occurred in people with asthma. As a result, regulatory authorities prohibited SCIT in the UK outside of clinics that were familiar with its use and had appropriate resuscitation facilities. In the United States, serious adverse events (AEs) were reported in patients with relatively mild disease, and severe reactions kept being reported until the end of the century (14). These concerns about safety and a need to perhaps simplify administration led to various improvements, and also to the development of SLIT.

### History of SLIT

Oral and sublingual route for the administration of allergen extracts was attempted in the 1900s, and the available vaccines were single allergen preparations (15); these efforts, however, failed to establish this method at the time. In the 1980s, several landmark studies kept demonstrating the safety and effectiveness of SLIT. Since 1986 there has been a revival of interest in SLIT.

Currently, there is no difference between the allergens used for SLIT and SCIT, although there are differences in the product quality requirements for each method (e.g., natural allergen extracts versus recombinant allergens) (16, 17).

Sublingual immunotherapy is now being used routinely in some parts of Europe (especially Italy and France) and is gradually spreading to Northern Europe and the United States. The introduction of SLIT could widen the scope of AIT and allow an increased number of patients to receive therapy (12).

## Mechanisms of Immunotherapy to Aeroallergens

With AIT, allergen extracts are presented to the immune system either subcutaneously (SCIT) or sublingually (SLIT). As the patient is already sensitized to the allergens in question, they react with a localized immune response. The allergens arrive in local lymph nodes either unbound *via* free diffusion or are taken up by dendritic or B cells (18). Breg cells, which also play a key role in the induction of immune tolerance to allergens, can suppress allergen-mediated inflammation through secretion of IL-10 and TGF-β. Thereby, effector T-cell responses are suppressed, and Treg cells are induced (19). Likewise, Breg cells might promote allergen tolerance through preferential production of IgG4 antibodies on differentiation to plasma cells. Further, B-cells produce IgG4 antibodies, which bind to the allergens without initiating a reaction, thus acting as “blocking antibodies” (20). In a recent study, the authors showed that Bregs were less prevalent in lungs of mice after allergen exposure confirming that the development of asthma alters the homeostasis of IL-10+ regulatory B cells, emphasize the importance of B cells in asthma, not only as IgE producers but also as suppressive cells able to constrain the pathological process (21). Additionally, Tregs cells suppress allergic responses directly and indirectly. They migrate from the site of their development in the lymph nodes back to the area of inflammation and release IL-10 and TGF-β, thereby reducing local inflammation (22). IL-10 can decrease B cell antigen-specific IgE production and increase IgG4 levels; reduce proinflammatory cytokine release from mast cells, eosinophils, and T cells; and elicit tolerance of T cells. As a consequence, responses to allergens are reduced after induction of regulatory T cells (23). The data also support the concept of a later allergen-specific immune deviation from a TH2 to a TH1 cytokine profile (24). Furthermore, Tregs suppress effector Th1/Th2/Th17 cells, allergen-specific IgE, mast cells/basophils/eosinophils; inhibit migration of effector T cells to tissues; and facilitate release of IgG4 (25).

**Figure 1:**
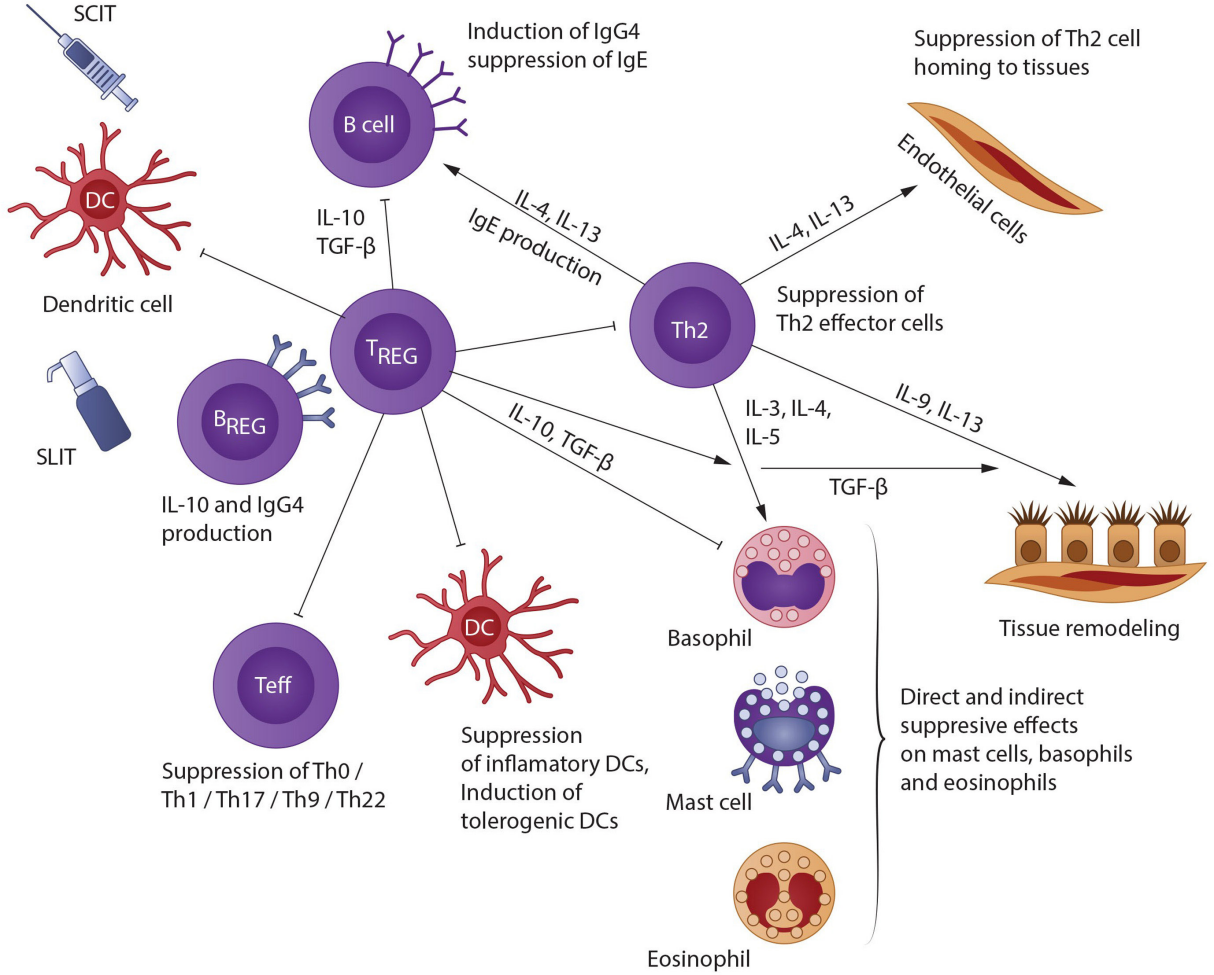
**A schematic representation of the mechanisms involved in AIT [modified from Akdis and Akdis (22)]**. Allergen IT results in both a shift in allergen-specific T-cells from Th2 to Th0/Th1, and in generation of IL-10- and TGF-β-producing T regulatory (Treg) cells. Treg cells affect B cells directly or indirectly by facilitating IgG4 and IgA release and hindering IgE development; also, they impede Th2 cell homing to tissues; they suppress mast cells, basophils, and eosinophils *via* direct and indirect mechanisms; and they inhibit epithelial cell activation. In addition, Breg cells also suppress effector T cells and contribute to IgG4 synthesis.

A schematic representation of the mechanisms involved in AIT is shown in Figure 1.

## Indications

Selection of patients for immunotherapy requires identification of the underlying antigenic trigger by combination of clinical history taking, and skin prick tests and/or blood tests for allergen-specific IgE (26). The current ARIA guidelines (27) give both SCIT and SLIT a conditional recommendation in allergic asthma, due to moderate/low quality of evidence. The majority of the guidelines agree that appropriate candidates for AIT are mainly children with allergic asthma that is difficult to control with conventional treatments. Asthma, nevertheless, must be well controlled by standard pharmacological treatment at the time the injection is administered, due to safety concerns (28). It is of particular interest in patients, who are sensitized to several pollens, to prescribe AIT only for major allergens (29), with the aim to increase the effectiveness of AIT and to better select patients who need a treatment. Hence, the use of molecular diagnosis techniques [component-resolved diagnostics (CRD)] (30) may allow physicians to better identify whether children with allergic respiratory symptoms are sensitized to major allergens or to cross-reactive molecules (31). In this context, an observational multicenter survey carried out by the Italian Pediatric Allergy Network suggest that a higher cutoff point of SPT-induced wheal reactions (e.g., 5 mm) should be used to take decisions when a confirmatory CRD assay cannot be implemented (32). In the UK, AIT is rarely used for asthma, partly because of the risk of adverse reaction with SCIT in uncontrolled asthma, and partly because of the lack of evidence for its cost-effectiveness versus the currently available routine treatments (33). Several factors may influence the decision for immunotherapy either way, such as poor adherence, clinically irrelevant allergens, poly-sensitizations, unavoidable adverse reactions of routine medication, etc. (34). Furthermore, the prescription of AIT depends also on the severity of the allergic asthma and duration of symptoms. Key parameters to evaluate the severity are the need of additional specialist visits; the response to pharmacotherapy and the recurrence of symptoms impairing school or sport activities or altering sleep quality (35). The decision between SCIT or SLIT hinges on several factors, including product availability, cost, patients ability to consistently attend the clinic, patient’s characteristics, physician’s/patient’s preference, etc. (4). Also, SLIT could be tried if SCIT causes systemic reactions (34). Indications for SCIT and SLIT are summarized in Table 1.

**Table 1:** **Indications and contraindications for subcutaneous immunotherapy and sublingual immunotherapy (SLIT) in asthmatic children**.

Indications	Contraindications
Mild–moderate allergic asthma, well or “partially” controlled by pharmacotherapy (53) Clinically relevant sensitization (82) Availability of a standardized product (28)	Malignant/cardiovascular/autoimmune disease Uncontrolled asthma Pregnancy Acute infections < 5 years old (36, 66, 82) Lack of compliance and severe psychological disorders (28) Inflammation, injury, or surgical intervention in oral cavity SLIT Acute gastroenteritis Eosinophilic esophagitis (39)

## Contraindications

The contraindications for SCIT or SLIT are either absolute contraindications (serious immunologic disease, major cardiovascular disease, cancer, chronic infections, lack of compliance, severe psychological disorders, etc.), or relative contraindications (pregnancy) (36). Severe asthma or uncontrolled asthma (regardless of its severity) is major risk factor for serious or even fatal adverse reactions and, therefore, represent important contraindications for SLIT/SCIT (37, 38). Interestingly, well-controlled asthma, regardless of its severity, was thought to *not* be a contraindication for AIT in a recent EAACI position paper (36). However, the strength of this recommendation was variable (36), and more evidence should become available before AIT can be safely considered for patients with severe asthma, even if well controlled. *Partially* controlled asthma is a *relative* contraindication for AIT in the same paper (36). Accordingly, German guidelines suggest that AIT may be performed in children with partially controlled asthma (39). Furthermore, well-controlled asthma, regardless of its severity, is not a contradiction for AIT (36). Any other condition that would reduce the patient’s ability to survive a potential systemic allergic reaction could also be a relative contraindication (28). SLIT should not be administered in case of acute inflammation, injury and surgical interventions in the oral cavity, or acute gastroenteritis (39). Some contradictions are listed in Table 1.

## Duration of Treatment

It is generally accepted that 3–5 years are required to achieve a clinical benefit and to maintain it after treatment cessation, for either SCIT or SLIT (39). Two studies showed no differences in the efficacy between a 3 and a 5 years course of house dust mite (HDM) in asthmatic children, or in the persistence of clinical benefit after discontinuation (40, 41). In another AIT study in asthmatic children sensitized to HDM, improvement has been shown from the first year of treatment (42, 43). The duration of the treatment may be prolonged (5 years or more), depending on the clinical response of subjects. Many patients experience a prolonged remission of symptoms after discontinuation of AIT (44, 45) whereas others may have a relapse of clinical manifestations. Currently, there are no specific laboratory tests or biomarker that can distinguish patients who will relapse from those who would have a prolonged clinical remission after discontinuing AIT (45). In keeping with that evidence, 3 years of SLIT in HDM sensitized children with asthma had a medication-sparing effect (46). The data are unclear, however, regarding the extend of the medication-sparing effect of AIT, with one study reporting no change in the asthma medication score after 1 year of treatment (43), whereas a pronounced effect was shown in a different work (42). Early treatment termination is a major problem (47) as only 35.4% of children were found to have completed at least 3 years of treatment (48). If AIT has been administrated for a number of years, current evidence suggests that it could induce long-term benefits, after its cessation (40, 46, 49). In any case, the duration of AIT should be individualized on the basis of the patient’s clinical response, disease severity, AEs, and patient preference (28).

Position papers and practice parameters recommend well-standardized protocols for SCIT in asthma. Pajno et al. showed that during the first year of SLIT for children with rhinitis/asthma because of grass pollen, the continuous regimen performed better than the pre/co-seasonal; however, no significant difference was shown in the subsequent 2 years (50). Currently, there is no clear evidence of superiority for the pre/co-seasonal for pollen allergens (11). Nevertheless, due to improved adherence and better cost-effectiveness, pre/co-seasonal regimens are often preferred (38).

## Efficacy

AIT is generally effective in asthmatic children who do not fully respond to asthma medication and environmental control. Nevertheless, it should be kept in mind that maintenance of asthma control *via* pharmacotherapy is vital both before and during AIT (51, 52). Several studies evaluating the efficacy of SCIT (53, 54) and SLIT (7, 55) have demonstrated effectiveness in controlling asthma symptom and reducing the medication use. A recent systematic review also concluded that SCIT and SLIT appear to be efficacious for the treatment of rhinitis and asthma in children (56).

### Efficacy of SCIT

There is consensus that SCIT for asthma induced by the most common aeroallergens (grass, mite, and cat dander) is generally efficacious (57). The efficacy of SCIT for the treatment of asthma, including a steroid—sparing effect, was evaluated in a meta-analysis including 101 studies (3,792 patients) carried out both in adults and in children (53). In particular, 42 studies of AIT involved patients with mite allergy, 27 pollen allergy (mostly grasses), 10 animal dander allergy, 2 *Cladosporium* allergy, 2 latex allergy, and 6 patients with multiple aeroallergens allergy. A significant reduction of symptoms was found in patients treated with mite and pollen AIT, while no significant improvement was recorded for animal dander or allergenic mixtures. Despite the heterogeneity of the included studies, the overall reduction of symptoms (for all allergens), the medication scores, and the bronchial hyperreactivity were significantly reduced, too. Saporta et al. evaluated 99 children and adults in regards to symptom score before and after either SCIT or SLIT. Coughing seemed to respond better to SCIT (*P* = 0.037), and wheezing to SLIT (*P* = 0.024), though both symptoms significantly improved regardless of regimen. For the remaining symptoms, there was no significant difference between SCIT and SLIT (58).

### Efficacy of SLIT

The evidence for clinical efficacy of SLIT is not abundant, but good efficacy is generally reported for HDM, and grass pollen allergens. A recent review found a relative efficacy of SLIT (symptoms and/or medication score) in adults and children from 20 to 40% (7). With respect to appropriate doses, the 300 IR (index of reactivity) dose of SLIT is thought to offer optimal efficacy and tolerability for HDM-induced asthma (59). A meta-analysis that included 9 studies on 441 asthmatic children found a significant decrease in symptom and medication scores with SLIT, in comparison to placebo (60). In another meta-analysis that evaluated 9 studies in 452 HDM-allergic children aged 3–18 years with asthma treated with SLIT, marked improvement in asthma symptoms and medication scores, and a steroid-sparing effect was seen (61). Overall, the reviews of the literature on pediatric populations consistently support the efficacy and safety of SLIT compared to placebo.

## Safety

In order to reduce the risk of adverse effects, AIT starts with very low doses that increase within the first few weeks to months of treatment (buildup/up-dosing phase), and until a maintenance dose is reached (28, 62). This does not, however, eliminate the risk of reactions, which is directly dependent on several factors such as allergen extract, injection schedule, dose, and patient factors (63). Such reactions could be local (in the immediate vicinity of the administration site) and systemic (SR), which can be further characterized as fatal, anaphylaxis, and systemic reactions not otherwise classified (wheezing and urticarial etch) (53). In most cases, symptoms can be managed if they are treated early.

The incidence of systemic reactions for AIT varies between 0.06 and 1.01% in those receiving SC dosing (64). In a recent prospective European survey, 762 children and 801 adolescents with AR (93.7%), AR and asthma (56.1%), and asthma alone (5.2%) had been included; they were sensitized to pollens (45%), mites (36.8%), dander (10.2%), or they were polysensitized (62.5%). A total of 29 reactions had been recorded, 23 by SCIT, and 6 by SLIT. The only three cases of anaphylaxis were related to SCIT, and they had a delayed onset (>2 h after administration) (65). Current recommendations suggest that children undergoing SCIT are observed for at least 30 min after injection (3, 66).

Typically, asthma is considered to be a risk factor for SRs, especially when it is uncontrolled (36). Other risk factors include polysensitization, grass pollen sensitization and—regarding SCIT—the use of natural extracts versus allergoids (65). All allergen preparations, such as standardized extracts (67), allergoids (68), or recombinant allergens (69), can cause side effects. Hence, research is being conducted to produce extracts using modified proteins or peptides that may increase safety and efficacy (33).

Sublingual immunotherapy appears to be quite safe for pediatric patients. In an observational study of 193 children receiving SLIT, who had a history of allergic rhinitis with or without asthma, there were nearly 500 mild/local adverse reactions but only 1 SR (severe asthma attack) (70). The main local AEs are oral/throat itching and mouth/tongue edema. In children, gastrointestinal complains have been mostly described during SLIT with HDM (7). Local symptoms can be, however, severe enough to warrant discontinuation of treatment. A grading system has been suggested with grade 1 corresponding to mild symptoms, grade 2 to moderate symptoms that require systemic treatment, and grade 3 to severe symptoms that could prompt termination of the SLIT regimen (7). The incidence of SRs with SLIT does not appear to be dose dependent, unlike SCIT where SRs are associated with higher allergen dose (71). A recent review summarized over 80 randomized double-blind placebo-controlled trials, and several reviews of both adult and children populations and concluded that, in most studies, the overall occurrence of systemic side effects is similar between placebo and active groups (7). To date, only few cases of anaphylaxis have been reported with SLIT (38), and some of these are probably due to overdose (72).

## Preventive Effect of AIT

Allergen-specific immunotherapy (AIT) is the only treatment capable of disease modification, as demonstrated by prevention of new sensitizations and inhibition of disease progression, especially in children monosensitized to HDM (73, 74). Due to its disease-modifying effects, AIT may be the closest that we currently have to a cure for allergic asthma (4). In the “Preventive allergy treatment (PAT) study,” SCIT with birch and/or grass pollen reduced the risk of asthma development in children with allergic rhinoconjunctivitis (75). This effect was detectable 7 years following discontinuation of SCIT (76).

Sublingual immunotherapy was also shown to have a preventive effect in a study in which 113 children, aged 5–14 years with seasonal rhinitis due to grass pollen, were randomly allocated to pharmacotherapy plus SLIT, or pharmacotherapy only. After 3 years, only 8 of 45 SLIT patients had developed asthma as opposed to 18 of 44 controls (confidence interval: 1.5–10) (77). In another trial, 216 children aged 5–17 who had rhinitis with/without intermittent asthma received conventional medication plus SLIT, or medication only. After 3 years of observation, the prevalence of persistent asthma was 1.5 and 30% for SLIT and the control group, respectively (78). In a further study, the same authors prospectively evaluated the long-term effect of SLIT in 59 patients, compared with 12 control subjects. The total duration of the follow-up was 15 years. All the control subjects developed positive tests to allergens previously negative, while this occurred in less than a quarter of the patients receiving SLIT (44). Zolkipli et al. could recently demonstrate a significant reduction in sensitization to new allergens in children prophylactically treated with SLIT. This was a prospective, randomized DBPC, proof-of-concept study involving 111 infants <1 year of age at high risk of atopy (positive atopic family history) with no sensitization to common allergens at randomization. After a year of treatment with a high-dose HDM SLIT, there was a 50% reduction in sensitization to any allergen in the active group (79).

## Special Considerations

### Age

Allergen immunotherapy for inhalant allergens is usually not considered for infants and toddlers. Although both SCIT and SLIT have been employed in children under 5 years and they appear to be effective (80), the evidence for the use of immunotherapy in this group is limited (81). For practical reasons, immunotherapy is not generally offered to patients below the age of 5, while for older ages there is no upper limit (82). In any case, each patient should be evaluated individually by considering the benefits and risks (83). SLIT drops are generally preferred for younger children over SCIT (48).

### Polysensitized Patients

According to the review by Calderon et al., 50–80% of patients with allergies are polysensitized. This impedes appropriate selection of patients for immunotherapy (37) and renders the clinical history vital in the identification of the clinically relevant allergen(s) (84). The use of *in vitro* component-based IgE diagnostics can increase the likelihood of AIT being successful, by facilitating correct identification of the culprit allergen (39). Multiallergen immunotherapy is currently supported by little evidence, both regarding its efficacy and successful induction of immunological tolerance (37). Also, there are conflicting results for the efficacy of allergen mixes (85). Thus, large clinical trials are needed before SCIT and/or SLIT can be routinely carried out with an allergen mixture or concomitant use of several allergens in polysensitized patients.

### Omalizumab and AIT

Omalizumab pretreatment has been shown to improve the safety and tolerability of cluster and rush immunotherapy schedules (86, 87). Additionally, omalizumab in combination with immunotherapy is more effective that AIT alone in managing symptoms (87). Treatment of >6 months with omalizumab was clinically effective in patients with severe uncontrolled asthma who could not tolerate immunotherapy (63, 88). This effect of omalizumab allowed the initiation of AIT in children with severe asthma. However, studies investigating AIT with omalizumab pretreatment and/or AIT-omalizumab combinations are lacking in children with severe asthma; further research is needed to evaluate the risk/benefit ratio of such regimens (63).

### SCIT vs SLIT

There is conflicting evidence regarding which method is more effective. Chelladurai et al. showed little difference in treatment effectiveness when comparing SCIT with SLIT (89). From four dust mite studies, two studies favored SCIT in reducing medication use and two favored SLIT, while a birch study found SLIT to be more effective (89). A meta-analysis by Nelson found that SCIT was superior to SLIT (90). In general, although both SCIT and SLIT appear to be effective in allergic asthma, literature is more supportive of an SCIT predominance in clinical efficacy (91).

In regards to safety, SLIT appears to be better tolerated than SCIT. The majority of SLIT AEs are local reactions (e.g., oromucosal pruritus) that appear at the start of treatment and resolve within a few days or weeks, without any medical intervention. Only, a few cases of SLIT-related anaphylaxis have been reported (92). A novel approach for AIT in which SCIT is administered in the buildup phase and SLIT in the maintenance phase in a randomized, controlled, prospective manner in HDM–sensitive asthmatic children was conducted. The novel regimen proposed seems to successfully combine the advantages of both routes without loss of clinical benefit and might be a promising alternative in children undergoing AIT (93).

## Other Issues

### Compliance

It is important that AIT is carried out in accordance with prescriber’s recommendations (2, 94). Adherence to therapy and the likelihood of treatment success are improved by thoroughly informing the patient about the way AIT works. Studies conducted on SCIT showed that the major cause of non-compliance was the inconvenience related to injections, and the cost of treatment (90). SLIT, on the other hand, had different compliance issues as it is administered at home by patients themselves. Although it was initially thought that SLIT would have a much better compliance than SCIT due to omitting the requirement to regularly attend clinics, it was soon shown that adherence to SLIT was not significantly better; this is probably because SLIT faces similar adherence problems with other conventional pharmacotherapy regimens (95).

### Cost-effectiveness

Studies comparing cost-effectiveness between patients treated for 3 years with AIT versus those treated with pharmacotherapy alone have found that AIT might be associated with cost savings as high as 80% 3 years after completion of treatment (4). Nevertheless cost-effectiveness is difficult to review due to different national health systems, variable epidemiologic data, and different prescription habits and outcome measures used in studies (96). However, in general, AIT’s cost-effectiveness appears to be good, as demonstrated by several pharmacoeconomics studies conducted within 6 years of treatment initiation (9).

## Conclusion

AIT appears to be effective in children with IgE-mediated asthma who do not fully respond to the conventional anti-asthmatic medications and environmental control and currently represents the only therapeutic approach capable to modify the natural evolution of a respiratory allergy. Its steroid-sparing effect is an important benefit for patients who have to use these drugs in high doses and in long-term regimens. Both SCIT and SLIT appear to be effective in allergic asthma, although some reports suggest that the efficacy of SCIT may be better. Uncontrolled asthma remains a significant risk factor for side effects, and AIT should not be considered on safety grounds for patients who cannot get their symptoms reasonably under control with pharmacotherapy alone. As we are entering the era of personalized medicine, further research should be conducted with a view to individualize AIT using recombinant antigen technology: this way we could perhaps create allergen extracts against specific proteins to which the patient is allergic, or extracts with modified proteins or peptides that could increase safety/efficacy. Adjuvants that can stimulate the immune system are currently being developed. These approaches have the potential to transform AIT to a mainstream, first line therapy in the foreseeable future.

## Author Contributions

ST had the conception and designed the work. ST, AM, and GF collected the data. All the authors contributed to the data analysis and its interpretation. ST and GG made the critical revision of the article, and GG gave his final approval of the version to be published.

## Conflict of Interest Statement

The authors declare that the research was conducted in the absence of any commercial or financial relationships that could be construed as a potential conflict of interest.
