# Corrigendum: Subcutaneous and Sublingual Immunotherapy in Allergic Asthma in Children

**DOI:** 10.3389/fped.2017.00187

**Published:** 2017-09-11

**Authors:** Sophia Tsabouri, Antigoni Mavroudi, Gavriela Feketea, George V. Guibas

**Affiliations:** ^1^Child Health Department, University Hospital of Ioannina, Medical School of Ioannina, Ioannina, Greece; ^2^Allergy Unit of the 3rd Pediatric Department, Aristotle University of Thessaloniki, Ioannina, Greece; ^3^Amaliada Hospital Unit, General Hospital of Ilias, Amaliada, Ioannina, Greece; ^4^Division of Infection, Immunity and Respiratory Medicine, University of Manchester, Manchester, United Kingdom; ^5^Allergy Department, University Hospitals South Manchester NHS Trust, Manchester, United Kingdom

**Keywords:** subcutaneous immunotherapy, sublingual immunotherapy, children, allergy, asthma

In the original article [Contraindications], paragraph 6, line 159, there was an error. The phrase “*Furthermore, well-controlled asthma, regardless of its severity, is not a contradiction for AIT*,” was contradicting other assertions in the “contraindications” paragraph. The following sentence should be disregarded: [*Furthermore, well-controlled asthma, regardless of its severity, is not a contradiction for AIT* (36).]

The authors apologize for this error and state that this does not change the scientific conclusions of the article in any way.

## Conflict of Interest Statement

The authors declare that the research was conducted in the absence of any commercial or financial relationships that could be construed as a potential conflict of interest.

